# Reproduction of the Solenopsis Mealybug, Phenacoccus Solenopsis: Males Play an Important Role

**DOI:** 10.1673/031.013.13701

**Published:** 2013-11-30

**Authors:** Fang Huang, Jing-Ming Zhang, Peng-Jun Zhang, Yao-Bin Lu

**Affiliations:** Institute of Plant Protection and Microbiology, Zhejiang Academy of Agricultural Sciences, Hangzhou 310021, China

**Keywords:** egg resorption, obligate amphimictic, parthenogenesis

## Abstract

The solenopsis mealybug, Phenacoccus solenopsis Tinsley (Hemiptera: Pseudococcidae), is an aggressive pest threatening crops worldwide. The biology of P. solenopsis has been described in several studies, but detailed information on the reproduction of P. solenopsis has not been investigated. The results of our study showed: 1) no progeny could be produced by virgins; 2) apoptosis of follicle cells, which occurs when the eggs begin to develop, did not happen in virgins; and 3) oosorption occurred in the unfertilized eggs. This suggests that P. solenopsis is an obligate amphimictic species, and resorption of developed eggs fits the “wait to reproduce” oosorption hypothesis. Compared to females that mated when they were two days old, the females that mated 30 days after eclosion had lower reproductive output and longer adult lifespans, but had higher reproductive output and shorter lifespan than those of the unmated females. Such a phenomenon suggests that resources obtained from eggs can be allocated for survival until conditions for reproduction improve. The results of this study provide evidence for a trade-off between survival and future reproduction: delayed reproductive conditions trigger physiological states geared toward survival at the expense of reproduction. The mating history of the males had no effect on progeny production.

## Introduction

The solenopsis mealybug, Phenacoccus solenopsis Tinsley (Hemiptera: Pseudococcidae), is polyphagous, having been recorded on plants of more than 100 genera belonging to over 50 families (Abbas et al. 2010), and recently emerged as a pest of cotton (Wang et al. 2010; Zhang et al. 2011; Huang et al. 2012). Cotton plants infested by P. solenopsis produce fewer bolls of smaller size with improper openings, and cotton seed yield is reduced by about half (Dhawan et al. 2007). Phenacoccus solenopsis has caused huge economic losses to cotton production in India and thereafter in Pakistan (ICAC 2011). It is thought that P. solenopsis become an aggressively invasive species on agricultural and ornamental plants, threatening the world cotton industry and other crops (Wang et al. 2010), due to considerable plasticity in morphology and adaptability in a wide variety of environmental conditions (Hodgson et al. 2008). It has been thus recently added to the European and Mediterranean Plant Protection Organization (EPPO) list (EPPO RS 2011/082) and Chinese quarantine pest list (AQSIQ 2009/1147).

Studies in Pakistan suggested that control of mealybugs with insecticides in the field has failed (ICAC 2011). Franco et al. (2004) suggested mating disruption could be considered in integrated pest management programs towards mealybugs as an alternative method to supplementary chemical treatments. Mating is the main stimulus for the oogenesis process and triggers the oogenesis process in insects (Davis 1964; Wheeler 1996), so its absence, or even its delay, may cause several modifications in the reproductive tract of the insects, including the oocyte (Bell and Bohm 1975), nurse, and follicle cells reabsorption (Martins and Serrão 2004; de Souza et al. 2007). Oosorption is defined as “a phenomenon in which developing oocytes are resorbed in the ovary in response to internal and/or environmental factors and is a specific strategy for reproduction that conserves resources and insures reproductive success” (Bell and Bohm 1975). However, parthenogenetic eggs in several insects, such as some species of aphids, need not such a trigger to activate the egg maturation (Blackman 1978).

Previous studies revealed that P. solenopsis females, through gamogenesis, have a high reproductive capacity and innate capacity for increase (Huang et al. 2011). Vennila et al. (2010) reported that parthenogenesis of P. solenopsis with ovoviviparity was dominant over the oviparous mode of reproduction. Further detailed information on the reproductive attributes of P. solenopsis is needed to clarify the reproductive behaviors and efficiency of P. solenopsis. Therefore, laboratory studies were performed on the following aspects of the bionomics of P. solenopsis: (1) survival rates of male-deprived and mated females; (2) patterns of ovarian maturation; (3) realized fecundity; and (4) the effect of age and mating-experience on reproduction.

## Materials and Methods

### Mealybugs and rearing

The reproduction of P. solenopsis was tested on its preferred host, cotton, Gossypium hirsutum L. (Malvales: Malvaceae). Colonies were maintained in rearing cages (50 × 50 × 50 cm) in the insectary at 27 ± 1° C and 60–80% RH, under a 12:12 L:D photoperiod. Plants of the same cotton cultivar (Zhefeng No. 1) were grown in plastic pots (13 cm diameter) with a mixture of peat moss, vermiculite, organic fertilizer, and perlite (10:10:10:1 ratio) in a climate room at 27 ± 1° C, 60–70% RH, and a 12:12 L:D photoperiod. All plants used in the experiments initially had 2–3 fully expanded true leaves.

The tested mealybugs were reared separately. One mealybug was held on a detached cotton leaf in a 50 mL Eppendorf tube with an opening (3–4 mm diameter) in the bottom side through which the leafstalk was dipped into a 1.5 mL Eppendorf tube half-full of water, which was replenished daily. A piece of organza material was used instead of the lid to allow ventilation for the tube.

### Adult longevity

Surviving females were recorded once daily and determined from 90 individuals.

### Ovarian maturation

To determine the rate of ovarian development and the number of eggs, females were dissected in saline solution, observed under a Nikon SMZ 1500 microscope (www.nikon.com) equipped with a Nikon digital sight DS-L1 camera. Fluorescent photos were taken under an Olympus BX 51 microscope (www.olympus-global.com) equipped with a QImaging Micropublisher 5.0 RTV camera (www.qimaging.com). Before photos were taken, the ovaries were freshly dissected and dyed with acridine orange (170 µg/mL) according to the method used by Hopwood et al. (2001). Ovaries were dyed for 1 min, and then cleaned with phosphate buffer saline (0.1 M, pH 7.0). Observations started from ≤ 24 hr after adult emergence and then every five days until all of the insects died (300 individuals total). Twenty individuals were dissected in every sampling stage. Microscopic observation showed that immature eggs of P. solenopsis were attached to nurse cells. According to the volume ratio of the nurse cells versus the oocytes, eggs were divided into three categories as the nurse cells became smaller. Type I: the ratio was > 1; type II: the ratio was < 1; type III: the ratio was near zero. Resorbed eggs were recorded as well.

### Fecundity

To determine reproduction of P. solenopsis, randomly selected unmated females, females that mated two days after adult emergence, and females that mated 30 days after adult emergence were individually reared. Progeny were counted once daily.

### Effect of males on reproduction

To determine the effect of mating on reproductive attributes, egg production of mated and unmated mealybugs were compared. Unmated females (50 individuals): late 3rd instar crawlers were carefully selected from the colony and transferred into the individual rearing tube to be kept alone for the remainder of their life. Mated females (50 individuals): each virgin female adult was selected and transferred into a Petri dish (3.5 cm diam.), and then one male adult was introduced. When mating was observed to be finished, the female was transferred into the individual rearing tube to be reared alone for the remainder of her life. Each male was used once.

### Effect of age on reproduction

Two-day-old adult females have been previously shown to be in a mating-ready state (Huang et al. 2011). To examine the effect of delayed mating, late 3rd instar crawlers (20 individuals × 3 replicates) were selected, and females were temporarily reared as virgins. Then, male adults were introduced 30 days after the adult emerged.

A previous study showed that adult longevity of mated P. solenopsis females was about 32 days (maximum 40 days) and for males was about four days (Zhu et al. 2010). To determine the effect of age on reproduction, egg production of two-day-old mated females and 30-day-old mated females were compared. Because males could be in a mating mode for about two days (Zhu et al. 2010), the effect of the age of males was ignored in this study.

### Effect of multiple mating on reproduction

To determine the effects of mating history on reproductive attributes, the effects of females and males mating multiple times (30 individuals × 3 replicates for both males and females) were considered. Males that mated multiple times: males with a mating experience on the previous day and virgin males were introduced to females.

Females that mated multiple times: the fecundities of females that mated once and females that mated twice were compared (the first mating was one day before the second mating). Males in both matings were virgin.

### Data analysis

Corresponding treatment means of adult female longevities and reproductive parameters (pre-oviposition period after mating, egg deposition, post-oviposition period, age at peak oviposition, progeny counts of peak oviposition, realized fecundity, and progeny counts of oviposition per day) were first checked to ensure the data could meet assumptions of normality and were then compared using ANOVA followed by Tukey's test.

## Results

### Adult longevity

The mean longevity of male-deprived P. solenopsis females was 65 ± 2.4 days, about twice that of the females that dated two days after adult emergence (33 ± 3.3 days). The mean longevity of the females that mated 30 days after emergence was 47 ± 2.3 days, which was significantly higher than that of the two-day-old females, but lower than that of the male-deprived females (p < 0.01). Virgin females that mated with males with previous mating experience had the same longevity as the females that mated when they were two days old females (p = 0.31). Mating multiple times did not significantly affect female longevity (*p* = 0.31).

### Egg maturation

**Figure 1.**
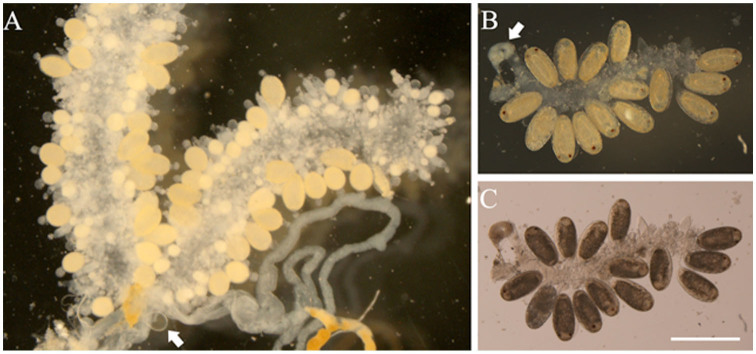
Photos of ovaries of unmated (A) and mated (B, C) Phenacoccus solenopsis females at 15 days post adult emergence. Arrows show the seminal vesicles. Bar = 500 µm. High quality figures are available online.

**Figure 2.**
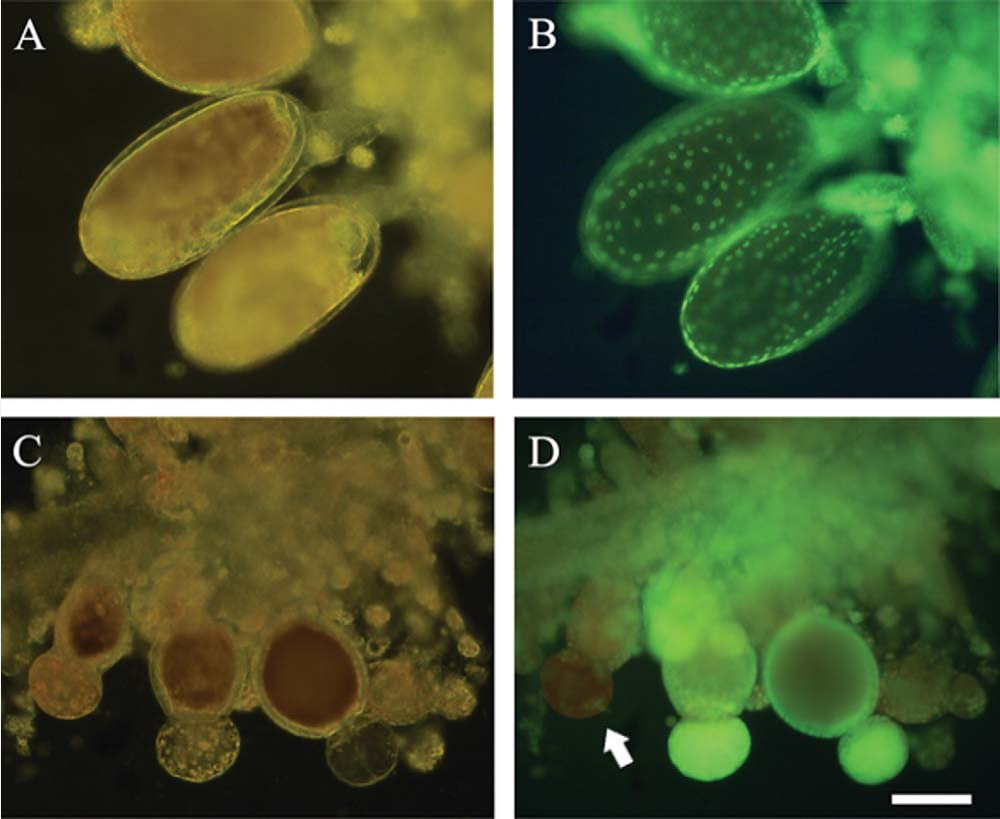
Photos of eggs in mated (A, B) and unmated (C, D) Phenacoccus solenopsis female ovaries. B, D: fluorescent photos of ovaries dyed by acridine orange. Arrow shows the resorbed egg. Bar = 100 µm. High quality figures are available online.

**Figure 3.**
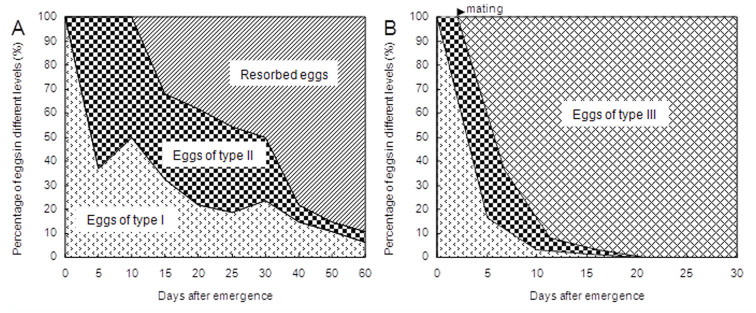
Ratios of eggs of different types from unmated (A) and mated (B) females, which were mated two days after adult emergence. High quality figures are available online.

Intact ovaries could be easily dissected from unmated females (Figure 1A); these were characterized by a transparent spermathecum (white arrow), and no egg developed beyond type III. By way of comparison, type III eggs, which had developed ommateum and body segments, were predominant in ovaries of mated females (Figure 1B). Their spermathecums were not transparent under the inverted microscope (Figure 1C). Fluorescent photos of ovaries from females that had mated showed follicle cells were undergoing apoptosis, as suggested by concentrated nuclei (Figure 2B). Such follicle cellular changes could not be observed in unmated females (Figure 2D). The development of eggs in unmated females ceased at type II; thereafter, the eggs were resorbed (white arrow in Figure 2D). In unmated females, egg resorption occurred 10 days after adult emergence (Figure 3A). In mated females, type III eggs were predominant 10 days after emergence, when the females started ovipositing (Figure 3B). The patterns of eggIntact ovaries could be easily dissected from unmated females (Figure 1A); these were characterized by a transparent spermathecum (white arrow), and no egg developed beyond type III. By way of comparison, type III eggs, which had developed ommateum and body segments, were predominant in ovaries of mated females (Figure 1B). Their spermathecums were not transparent under the inverted microscope (Figure 1C). Fluorescent photos of ovaries from females that had mated showed follicle cells were undergoing apoptosis, as suggested by concentrated nuclei (Figure 2B). Such follicle cellular changes could not be observed in unmated females (Figure 2D). The development of eggs in unmated females ceased at type II; thereafter, the eggs were resorbed (white arrow in Figure 2D). In unmated females, egg resorption occurred 10 days after adult emergence (Figure 3A). In mated females, type III eggs were predominant 10 days after emergence, when the females started ovipositing (Figure 3B). The patterns of egg maturation were the same in females that mated multiple times (data not shown).

### Effect of male/age/multiple matings on fecundity and reproduction

**Figure 4.**
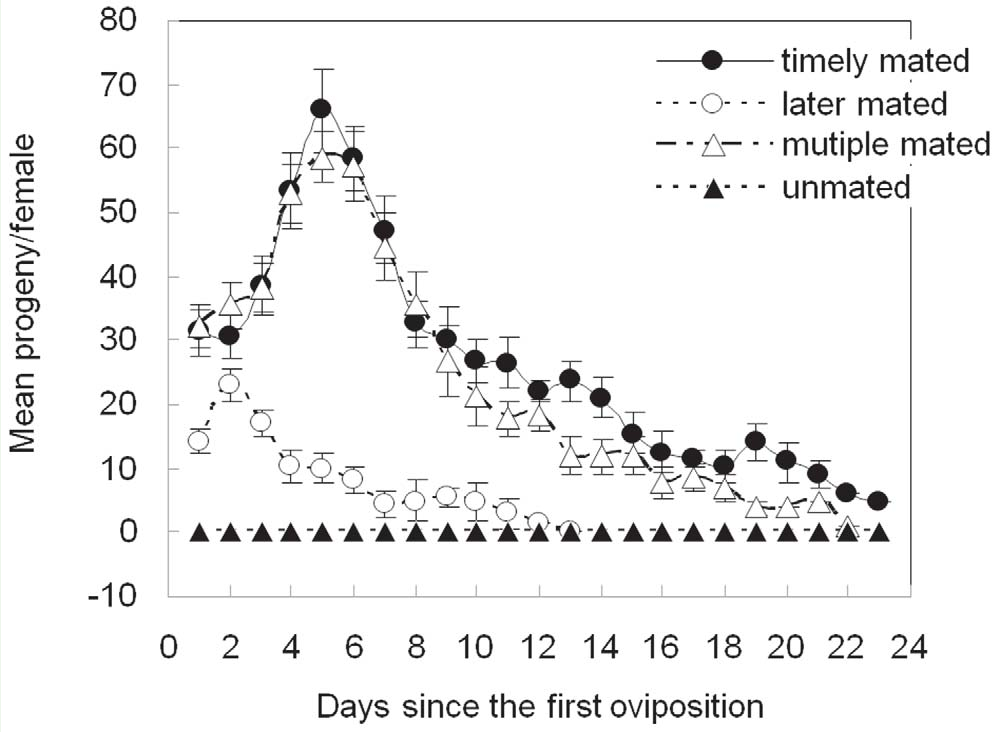
Mean realized fecundities of Phenacoccus solenopsis females that mated two days after adult emergence, 30 days after post emergence, not at all, and multiple times. Error bars represent ± SEM. Timely mated = mated two days after adult emergence. Later mated = mated 30 days after adult emergence. Multiple mated = mated multiple times. High quality figures are available online.

Unmated females had no progeny (Figure 4). The fecundity of females that mated when they were two-days-old and the fecundity of females that mated multiple times was characterized by an early peak followed by a decrease, and the two patterns were similar. The pattern of fecundity from females that mated 30 days after emergence also had an early peak and then a gradual decrease, but the progeny counts were significant lower compared to the females that mated after two days (*p* < 0.01).

**Figure 5.**
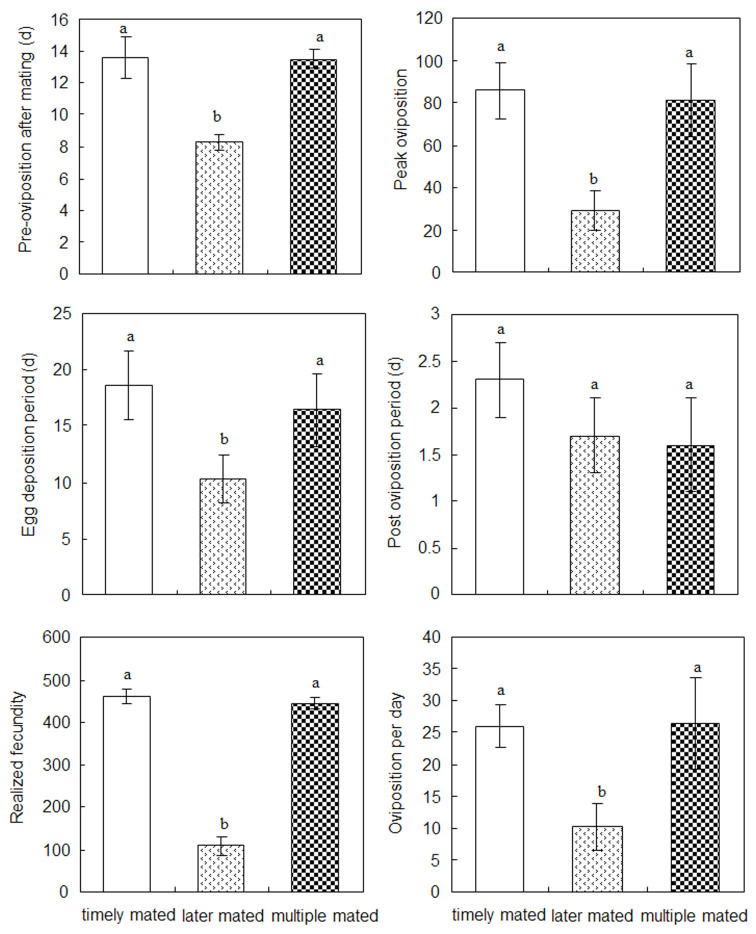
Reproductive attributes of female Phenacoccus solenopsis from different mating histories, i.e., mated two days after adult emergence, mated 30 days after adult emergence, and mated multiple times. Error bars represent ± SEM. Different letters above bars indicate a significant difference at *p* < 0.05 as determined by ANOVA followed by Tukey's test. Timely mated = mated two days after adult emergence. Later mated = mated 30 days after adult emergence. Multiple mated = mated multiple times. High quality figures are available online.

Compared to the reproduction of two-dayold mated females, the reproduction of females that mated multiple times showed no significant differences (p > 0.05), and mating multiple times had no significant effect on reproductive characteristics (Figure 5). However, several parameters of females that mated 30 days after emergence, such as preoviposition period after mating, egg deposition period, progeny count at peak oviposition, fecundity, and progeny produced per day, were significantly lower than those of females that mated two days after emergence or females that mated multiple times (Figure 5).

## Discussion

Most species of scale insects (Hemiptera: Coccoidea) reproduce sexually (Gullan and Kosztarab 1997), although several types of parthenogenesis have been described in coccoids, including obligate and facultative parthenogenesis (da Saliva et al. 2010). Hermaphroditism has been reported only in Iceryini (Margarodidea) (Normark 2003).

The genetic sex determination systems of scale insects belong to haplodiploidy or thelytoky (da Saliva et al. 2010). Facultative parthenogenesis is typically amphimictic, but unmated females may produce some viable offspring by thelytoky (Normark 2003). This type of reproduction in mealybugs is controversial, as several reports of facultative parthenogenesis were shown to be obligate amphimictic, such as in Planococcus citri, Pseudococcus calceolariae, and Pseudococcus viburni (da Saliva 2010). In P. solenopsis, amphimixis was confirmed in by Huang et al. 2011.

In this study, no progeny from unmated P. solenopsis females was recorded, even when the adults had different population densities or temperatures (data not shown). But parthenogenesis was suggested to be the dominant reproductive mode of P. solenopsis in open fields (Vennila et al. 2010). During the field-sampling in our study, few males were visible. But, when samples of plant tissues infected with mealybugs were immersed in 75% ethanol solution, male bodies could be found upon close inspection. In the laboratory rearing, actively flying males searching for mates could mainly be observed during 08:00–08:30. After 09:00, all males were concealed. Such a behavioral phenomenon might lead to an apparent absence of observations of adult males in the fields, which could mislead one to suggest parthenogenesis. However, several hemipteran insects show alternative reproductive modes, i.e., one species could have two reproductive avenues (either facultative or cyclical) (Normark 2003). For example, the grain aphid, Sitobion avenae, displays variations of its reproductive mode in response to host plants and temperature, which lead to different modes of reproduction between the same species from two regions (Sunnucks et al. 1997). Thus, it is possible some factors differing between China and India might also account for the apparent differences in the reproductive mode of P. solenopsis.

The development of type II eggs ceased in virgin females, followed by an increased maternal longevity, which suggests that oosorption in virgin female P. solenopsis was occurring. This ability to degrade unfertilized oocytes and resorb their nutrients is proposed to be an adaptive mechanism to optimize fitness in hostile environments that may lack mates, food, or host substrate (Bell and Bohm 1975; Barrett et al. 2008). In such an environment, reproduction is unlikely to be successful, so resources invested in oocytes can be recouped and reinvested into somatic functions that increase lifespan (Bell and Bohm 1975; Burger et al. 2004). Such a nutritional rearrangement in P. solenopsis females between maintenance and reproduction could be regarded as an adaptive response to a male-absent condition for an obligate amphimictic reproductive system. It is presumed that the resources resorbed from eggs can then be allocated to survival until conditions for reproduction improve (Bell and Bohm 1975). The data from our study show that harsh reproductive conditions triggered a physiology state geared toward survival at the expense of reproduction. A previous study revealed that females that mated when they were two days old survived about 30 days (Huang et al. 2011). Females that mated when they were 30 days old could produce progeny after mating, but the numbers of offspring were significantly less than those of the females that mated when they were two days old, and they had a significantly shorter lifespan than the unmated females. On the other hand, it could be suggested that males can strongly influence the germ/soma balance and alter reproduction/ longevity tradeoffs. Such reproductive tradeoffs under harsh environments have been reported in many other insects, e.g., beetles (Kajita and Evans 2009), bugs (Kotaki 2003), mosquitoes (Clifton and Noriega 2011), moths (Wang et al. 2011), and parasitoids (Guo et al. 2011).

Compared to no eggs laid by unmated females, an increase in the number of eggs produced by females that mated after 30 days was mirrored by a reduction in eggs laid by resorption of eggs that developed to type II. Thus, the females seemed to be “raiding the oocyte larder” for resources rather than constraining future reproductive potential through apoptosis in the new oocytes (Moore and Attisano 2011). Such a schedule of egg maturation fits the “wait to reproduce” assumption of the oosorption hypothesis.

The oosorption hypothesis is underpinned by the “Y model” (van Noordwijk and de Jong 1986; King et al. 2011), in which negative correlations among traits such as reproduction and longevity and current versus future reproduction arise through competition for limiting resources (Moore and Attisano 2011). Most studies of oosorption in insects thus focus on host plant availability and quality (Awmack and Leather 2002). In our study, an absence of mating opportunities was the limiting resource instead of food or host stress. The results demonstrate that under mating stress, oosorption could also occur; the superfluous nutrients redirected from eggs led to a longer adult survival.

Multiple male copulations are suggested to have detrimental effects on female fitness in terms of fewer sperm, fewer nutrients from nuptial gifts, prolonged duration of the copulation, increased risk of unsuccessful fertilization, or shorter subsequent lifespan (Elzinga et al. 2011). The results of our study showed that mating history had no effect on progeny production. The solenopsis mealybug females are immobile, while the males are short-lived and do not feed (Zhu et al. 2010). Due to its obligate amphimictic reproductive traits, mating multiple times is expected to lead to some limitations in both females and males, which we plan to explore. If only the obligate amphimictic reproductive pattern of P. solenopsis is unequivocally demonstrated, semiochemistry of this species would be our next studying target, which would be a possible guide for using mating disruption in integrated pest management programs.
